# Immunological Control of Viral Infections in Bats and the Emergence of Viruses Highly Pathogenic to Humans

**DOI:** 10.3389/fimmu.2017.01098

**Published:** 2017-09-11

**Authors:** Tony Schountz, Michelle L. Baker, John Butler, Vincent Munster

**Affiliations:** ^1^Arthropod-Borne and Infectious Diseases Laboratory, Department of Microbiology, Immunology and Pathology, Colorado State University, Fort Collins, CO, United States; ^2^Australian Animal Health Laboratory, Health and Biosecurity Business Unit, Commonwealth Scientific and Industrial Research Organisation, Geelong, VIC, Australia; ^3^Department of Microbiology, Carver College of Medicine, University of Iowa, Iowa City, IA, United States; ^4^Virus Ecology Unit, Rocky Mountain Laboratories, National Institutes of Health, Hamilton, MT, United States

**Keywords:** bats, Chiroptera, zoonosis, antibody repertoire, emerging infectious disease, virus ecology

## Abstract

Bats are reservoir hosts of many important viruses that cause substantial disease in humans, including coronaviruses, filoviruses, lyssaviruses, and henipaviruses. Other than the lyssaviruses, they do not appear to cause disease in the reservoir bats, thus an explanation for the dichotomous outcomes of infections of humans and bat reservoirs remains to be determined. Bats appear to have a few unusual features that may account for these differences, including evidence of constitutive interferon (IFN) activation and greater combinatorial diversity in immunoglobulin genes that do not undergo substantial affinity maturation. We propose these features may, in part, account for why bats can host these viruses without disease and how they may contribute to the highly pathogenic nature of bat-borne viruses after spillover into humans. Because of the constitutive IFN activity, bat-borne viruses may be shed at low levels from bat cells. With large naive antibody repertoires, bats may control the limited virus replication without the need for rapid affinity maturation, and this may explain why bats typically have low antibody titers to viruses. However, because bat viruses have evolved in high IFN environments, they have enhanced countermeasures against the IFN response. Thus, upon infection of human cells, where the IFN response is not constitutive, the viruses overwhelm the IFN response, leading to abundant virus replication and pathology.

Bats have gained attention in recent years as reservoir or suspected reservoir hosts of many high-impact human pathogenic viruses that cause outbreaks and epidemics with high mortality (1, 2). In terms of viral species richness and zoonotic potential, bats may be the most important mammalian sources (3, 4). Each of these viruses, including the ebolaviruses, Marburg virus, severe acute respiratory syndrome and Middle East respiratory syndrome coronaviruses, rabies and other lyssaviruses, and Hendra and Nipah viruses, is thought to circulate in certain species of bats without significant disease. Chiroptera, to which bats belong, is the second largest mammalian order, with about 1,200 species. Bats originated about 80 million years ago (mya) and substantial radial divergence ensued soon after the K–T extinction event about 66 mya (5). Consequently, bats have been on independent evolutionary trajectories for most of the history of mammals. They belong to the mammalian superorder Laurasiatheria that includes ungulates and canines, whereas rodents and primates belong to the superorder Euarchontoglires; these superorders diverged about 90 mya. Genome and transcriptome analyses suggest the immune systems of bats are substantially similar to those of other mammals; however, there are some significant differences, including the loss of the PYHIN locus that has the AIM2 cytosolic DNA sensor and inflammasome genes, loss of killer cell immunoglobulin-like (KIR), and killer cell lectin-like (KLR) receptor loci used by NK cells, expanded immunoglobulin heavy-chain VDJ segments and contraction of the interferon-α (IFNα) locus (6–11). Although bats share many immunological features with other mammals, little research into their immune systems or responses has been conducted and there are no well-developed bat research models to study infectious agents (12, 13).

Often, in zoonotic virus/reservoir host relationships, which have been best studied in rodents and primates (14–16), each virus is hosted by individuals of one or only a few species. There are exceptions, including slowly replicating viruses, such as rabies virus. However, viruses, like all other biological entities, are subject to the pressures of evolution and are likely genetically and biochemically adapted (“optimized”) to circulate within their reservoir host populations to either cause persistent infection (often for the life of the host), or to replicate and be shed for a sufficient period to allow transmission to other susceptible hosts, without causing substantial disease within the population (17). They typically do not elicit robust immune responses in their reservoirs, which could lead to viral clearance or immunopathology. When spillover of pathogenic viruses to humans or other non-reservoir species occurs, they are not biochemically optimized for the new host cells, which can lead to disease and death, or immune clearance.

Because of the occurrence of severe human diseases caused by some of the bat-borne viruses, an important question is; how do bats host these viruses without becoming diseased? The answer to this question is likely complicated and will vary between species of bats and species of viruses. In rodent reservoirs of pathogenic hantaviruses, in which the viruses establish persistent infection without meaningful pathology (18–22), the immune response is slow to develop (21) and is mediated by Fox-p3^+^, TGFβ-expressing regulatory T (Treg) cells, which counter inflammatory disease (23, 24) but at the expense of sterilizing immunity. Do bats have Treg cells? If so, do bat viruses also elicit Treg responses in their reservoir hosts? T cell genes are found in bats, but there are no publications demonstrating antigen-specific T cell activities in bats. The lack of such studies underlies a significant deficit in the study of bat immune responses, considering the functional subsets of T cells that have been identified in other species (e.g., Th1, Th2, Th17, NKT, Tfh, CTL, etc.) and the effector functions mediated by T cells, including T cell help, inflammation, chemotaxis, and augmentation of macrophage activities such as phagocytosis and killing of microbes.

Even less is known about NK cells in bats. Does the loss of KIR/KLR genes in bats (8) mean that NK cells use alternative receptors to recognize MHC class I for activation and inhibition? Do bat NK cells have the same effector functions found in other species, such as granzyme-mediated cytolysis and antibody-dependent cell cytotoxicity? Genes for granzymes A and B and CD16 (FcRγIII) are found in bats (6, 7); thus, it is likely that bat NK cells are functionally similar to other species in this regard. Until methods are developed to assess T cell and NK cell functions in bats, our understanding of bat virus infections of reservoir hosts will be severely limited.

Pathogenic bat-borne viruses encode immune modulating accessory proteins that often target the innate antiviral responses of infected cells. It is thought that these proteins are contributory factors of human disease (“virulence factors”) (25–32); however, because they evolved in their bat reservoirs (i.e., biochemically optimized), their impacts on the orthologous proteins of humans must somehow be different; otherwise, there would not be differential outcomes in bats (no disease) and humans (disease).

## The “Flight as Fever” Hypothesis

One proposed explanation for the lack of disease in virus-infected bats is the “flight as fever” hypothesis that suggests elevated body temperature during flight somehow mimics the effects of the fever response (33). However, the fever response after infection is much more sophisticated than simply elevated body temperature. In other mammals that have been studied, the production of interferons (IFN), interleukin-1, and prostaglandins have already occurred by the time fever is detectable (34, 35). There is no evidence that these effector molecules are expressed by bats during flight. Moreover, viral infections are complex processes; thus, it is unlikely that elevated body temperature alone is sufficient to explain how bats can host these viruses without signs of disease. The only experimental work assessing this effect showed that increasing incubation temperature of bat cells does not affect their ability to support ebolavirus replication (36). Although there is no experimental evidence supporting the flight as fever hypothesis, some have speculated this provides a metabolic mechanism for bats to host these viruses without disease (37).

## The “Always on” IFN System of Bats

An interesting feature of pteropid bats is that parts of the type I IFN system appear to be constitutively active and it has been hypothesized that this “always on” activity may hamper early viral replication (10). We have also observed signatures of IFN receptor pathway activation in uninfected primary kidney cells cultured from Jamaican fruit bats (*Artibeus jamaicensis*), including constitutive STAT1 phosphorylation (unpublished data). In other mammals that have been examined, the type I IFN loci have undergone expansion by tandem duplication events, leading to multiple copies of *Ifna* genes. However, in bats there is compelling evidence that the type I IFN locus has undergone contraction leading to fewer *Ifna* genes (10). Despite this contraction, *Ifna* basal gene expression in bats is elevated relative to humans and rodents, as are the levels of many IFN-stimulated genes (ISG). The constitutive *Ifna* expression appears to induce a profile of ISGs that is not inflammatory, and this may be one of the reasons why *Ifna* expression can be elevated without leading to chronic inflammatory pathology. Furthermore, as the levels of IFNα protein expressed by bat cells remains to be determined, it is possible that much of the *Ifna* mRNA remains untranslated, providing a source of transcripts for rapid translation when required. In addition, the type III IFN response appears to be restricted to immune cells and epithelial cells, and can be activated independently of type I IFN signaling (38, 39). Collectively, these aspects of the innate immune system suggest that bat cells are poised to respond to viral infections immediately, which may restrict, but not prevent, viral replication. It is unknown why bat IFN pathways are constitutively active but some species of bats can have extraordinarily high population densities with extensive mutual grooming behavior. Because transmission of infectious agents is related to population density, it may be evolutionarily favorable for bats to hamper virus replication and shedding to limit transmission within a population. However, viruses must also be able to sufficiently evade the response to transmit within the bat population; otherwise, they would be driven to extinction. Thus, it is likely that bat virus accessory proteins are finely tuned to modulate bat antiviral responses.

## Immunoglobulin Repertoires of Bats

The germline immunoglobulin loci of mammals contain tandem variable (V), diversity (D), and joining (J) gene segments that recombine during B cell development in the bone marrow to generate VDJ rearrangements at the immunoglobulin heavy-chain locus, and VJ rearrangements at the light chain locus (40). The number of segments varies between species (Table 1), and not all segments are functional. For example, humans have 87 immunoglobulin heavy-chain V segments, but only about 40 are functional. V(D)J recombination generates the naive B cell immunoglobulin repertoire of an individual with, in humans, about 2 million unique immunoglobulin specificities that typically have an IgM heavy chain (41). The result, termed *combinatorial diversity*, occurs prior to, and is independent of, the antigen stimulation of an immune response. Swine, on the other hand, have far fewer heavy-chain gene segments, with seven functional V segments, two D segments and one J segment for just 14 possible combinations (42). Increased junctional diversity of the developing naive immunoglobulin repertoire occurs during recombination of the V(D)J segments in which exonuclease activity removes nucleotides from the segment ends and the enzyme *terminal deoxynucleotidyl transferase* (TdT) adds nucleotides to the segment ends (41). The substantially limited swine VDJ is overcome by exonuclease and TdT activities (42).

**Table 1:** Immunoglobulin gene segments of select mammalian species (42).

Species	V_H_ (*F*^a^)	D_H_	J_H_	V_λ_ (*F*)	J_λ_	C_λ_^b^	V_κ_ (*F*)	J_κ_	C_κ_	k:l^c^
Little brown bat	>250 (5)	?	13	?	?	?	? (?)	?	?	?:?
Human	87 (7)	30	9	70 (7)	7	7	66 (7)	5	1	60:40
Mouse	>100 (14)	11	4	3 (3)	4	4	140 (4)	4	1	95:5
Rabbit	>100 (1)	11	6	? (?)	2	2	>36 (?)	8	2^d^	95:5
Horse	>10 (2)	>7	>5	25 (3)	4	4	>20 (?)	5	5	5:95
Cattle	>15 (2)	3	5	83 (8)	>2	4	? (?)	?	1	5:95
Swine	>20 (1)	2^e^	1^e^	22 (>2)	>4	4	14–60 (5)	5	1	50:50

*^a^Number of families (*F*) of variable region genes*.

*^b^J_λ_–C_λ_ duplicons are the common motif in most mammals*.

*^c^Ratio of expressed light chain in adults expressed as percent*.

*^d^Rabbits have a duplicate of the entire kappa locus*.

*^e^Functional D_H_ and J_H_ genes*.

*?, number is unknown*.

Antigen exposure to naive B cells leads to secretion of IgM antibodies with typically low average affinities (~10^−7^ *K*_d_). This low affinity is the result of the poor proximity of the amino acid residues of the antibody variable region to the residues of the antigenic epitope, thus, fewer non-covalent bonds can form at the antibody:antigen interface. However, as these B cells undergo clonal selection and expansion during an infection, *somatic hypermutation* (SHM) occurs in daughter cell V(D)J regions that leads to antibodies with higher average affinities by virtue of refined complimentary topology between the antibody and its epitope, and the inclusion of amino acids in the variable region that strengthen the non-covalent interactions with the epitope (43). This process, termed *affinity maturation*, requires T cell help and expression of the enzyme *activation-induced cytidine deaminase* (AID) in the dividing B cells. The result of this process is the generation of antibodies with affinities for antigen that are orders of magnitude greater (~10^−10^ to ~10^−12^ *K*_d_) than those of the original naive parental B cell clones that recognized the antigen.

Affinity maturation can take weeks, but if the host survives the infection it typically produces antibodies that can bind antigen with nearly irreversible affinities under physiological conditions. Importantly, this process leads to memory B cells that have high affinity surface immunoglobulin receptors that have already class-switched to IgG or IgA, and can rapidly divide and secrete antibodies independent of T cell help should the same pathogen be encountered again. Indeed, affinity maturation principally accounts for the high antibody titers detected by the various serological end-point dilution assays.

**Figure 1:**
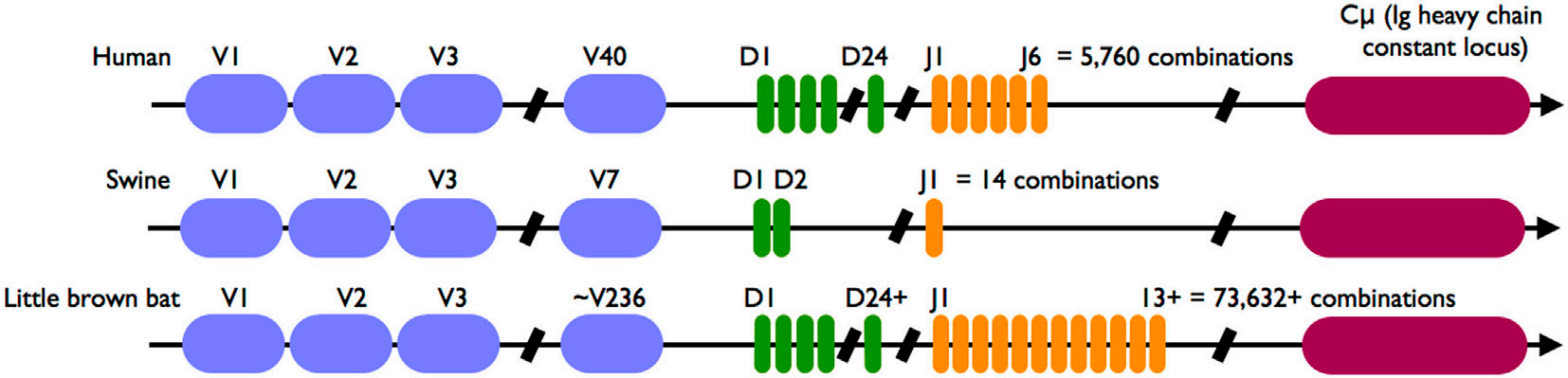
Immunoglobulin VDJ combinatorial diversity potential of humans, swine and little brown bats. The human heavy (H) chain locus has about 40 variable (V), 24 diversity (D) and 6 joining (J) segments that are functional. Rearrangement of the locus occurs during B cell development in the bone marrow and provides the repertoire of immunoglobulin specificities that are clonotypic in naive B cells, usually with IgM constant (Cμ) heavy chains, which populate peripheral lymphoid tissues, such as lymph nodes and the spleen. The theoretical number of human H chain specificities is 40 × 24 × 6 = 5,760. In contrast, swine have 7 V, 2 D, and 1 J segments that are functional, totaling 14. In the little brown bat (*Myotis lucifugus*), the functional H chain locus is estimated to have 236 V segments, at least 24 D segments, and at least 13 J segments, for a theoretical number of at least 73,632 specificities in the naive B cell repertoire. Blue, V gene segments; green, D gene segments; orange, J chain segments; purple, C gene (IgM); other C genes not shown.

In bats, combinatorial diversity may lead to the generation of a much larger naive immunoglobulin repertoire than it does in humans because bats may possess more heavy-chain VDJ germline gene segments. The heavy-chain locus of humans has 40 functional V segments, 24 D segments, and 6 J segments for a potential of 5,760 H chain specificities in its naive B cell repertoire through combinatorial diversity (44). The little brown bat (*Myotis lucifugus*) has an estimated 236 V segments, at least 24 D segments, and at least 13 J segments, with a potential of more than 70,000 specificities in the naive B cell repertoire (9) (Figure 1). Pteropid bats also have a highly diverse genomic and expressed V_H_ repertoire, and evidence for multiple expressed D_H_ and J_H_ segments, consistent with their ability to recognize a range of antigenic epitopes (45). Bats appear to only express λ light chains but no research has been published about their light chain VJ segments nor their T cell receptor genes, thus it is not possible to compare them to other mammals.

In little brown bats, there appears to be less dependence on affinity maturation (9), suggesting the expanded repertoire from combinatorial diversity may have reduced, but not eliminated, the evolutionary need for SHM. Bats have and express the gene for AID (6–8), thus it is likely that some SHM occurs but AID appears to play a less prominent role in bats than it does in mice, humans, and swine. AID also facilitates class switching to other antibody classes (isotypes) in mice and humans, such as the IgG subclasses and IgA (46). If it is used less in bats, does this mean there is less class switching in bats? Or is it only the SHM function that is reduced? Do bats also generate junctional diversity through exonuclease and TdT activity? If bats do not use SHM does that lead to less robust memory B cell responses that can contribute to viral recrudescence?

Many of these questions are difficult to address because there are few immunoglobulin class-specific antibodies for bats of any species. Polyclonal rabbit antibodies to Australian black flying fox (*Pteropus alecto*) IgG, IgM, and IgA have been generated (47), but all others are polyclonal antibodies to whole bat IgG that likely recognize light chains as well as heavy chains. Because light chains are shared by all immunoglobulin classes, these polyclonal antibodies cannot discriminate IgD, IgM, IgG, IgA, or IgE. The number of immunoglobulin heavy-chain genes that encode IgG subclasses vary between species, with Seba’s fruit bat (*Carollia perspicillata*) having only one, big brown bat (*Eptesicus fuscus*) having two, greater short-nosed bat (*Cynopterus sphinx*) having three and little brown bat having five (48). One monoclonal antibody to the immunoglobulin λ chain of the big brown bat has been generated and it has cross reactivity to little brown bat λ chains; thus, it likely will be useful for characterizing antibodies from these bats (49).

A recent report suggested that IgG, rather than IgA, is more abundant in mucosal secretions of the Australian black flying fox (47). Their approach used Jacalin, peptide M, and staphylococcal superantigen-like protein 7 to purify IgA; these reagents are routinely used to purify human IgA but it is unclear if they bind bat IgA efficiently, or at all. The suggestion that IgG is the most abundant secretory immunoglobulin in bats is inconsistent with established data on secretory immunoglobulins in other mammals and should be regarded with caution. The generation of isotype-specific monoclonal antibodies to bat immunoglobulins will be required to determine which isotype is most abundant in mucosal secretions of bats.

## A Hypothesis of Bat Immune Responses to Viral Infections

We propose that innate and adaptive immune responses in bats are different than in mice and humans, and these differences may account for why the viruses they host can be significant human pathogens. First, we suggest that because of higher constitutive expression of IFNα and persistent ISG activity, bat cells hamper virus replication relative to what occurs in cells from humans and rodent disease models. Second, because of the apparently larger bat naive immunoglobulin repertoires (from combinatorial diversity), bats may have more immunoglobulin specificities that favor clonal selection of B cells with immunoglobulin receptors that interact with substantially higher mean affinities for antigen. Because of this, and because of the reduced viral burden due to persistent IFN activity, there has been less evolutionary pressure for SHM to control viral infections in bats—the selected B cells can undergo clonal expansion without an urgent need for affinity maturation to generate high-titered antibodies.

This combination of events may lead to several expected outcomes that can be experimentally tested. First, because of their constitutively high expression of IFNα, less virus replication should occur in infected bat cells compared to human or rodent cells used in pathology models. Second, because of the hampered viral replication, antibody responses in bats likely are slower to develop and may not be as durable as those in mice or humans because fewer viruses will be available for T and B cell stimulation. Third, antibody titers, which are a function of affinity maturation, should be lower in bats (e.g., average affinities of 10^−9^ to 10^−11^ *K*_d_) than those generated in mice. A poorer antibody response (i.e., lower titer) could prevent or delay clearance of virus from the reservoir bat, contribute to persistent infection, prolong shedding, and lead to periods of recrudescence as antibody wanes.

Although there are a few bat cell lines that are susceptible to these viruses, caution must be exercised when using them because of the potential for unusual genomic events that routinely occur during immortalization, such as deletions and duplications of genes, which can complicate interpretation of data. Therefore, assessment of viral infection kinetics will require isolation of identical susceptible primary cells from reservoir bats, and humans or rodents used in pathology studies. As one example, Nipah virus infects endothelial cells (50–52) and the Syrian hamster is a pathology model for its disease (53, 54). Isolation of primary endothelial cells (e.g., PCAM1^+^ cells) from pteropid reservoir hosts and hamsters could be used to assess virus replication kinetics. If our hypothesis is correct, then we would expect less virus replication and shedding from primary pteropid endothelial cells than from hamster endothelial cells.

Consistent with our hypothesis, bat antibody responses appear to be slower to develop and less robust during infection (55, 56) and immunization (57, 58) compared to those of mice. Immunization of Brazilian free-tailed bats (*Tadarida brasiliensis*) and Egyptian fruit bats (*Rousettus aegyptiacus*) with rabies vaccines resulted in neutralizing antibody responses that are considered protective (59, 60). However, the vaccines used in these studies were inactivated, thus (1) were incapable of infecting cells and influencing the IFN response with the viral accessory proteins and (2) were formulated with adjuvant that simulates inflammation that contributes to more robust antibody responses. Experimental Marburg virus infection of Egyptian fruit bats, a natural reservoir host, leads to brief viremia, wide tissue distribution and low to modest viral loads and seroconversion (61–63) and transmission (64). Similarly, poor neutralizing antibody responses occur after experimental infection of artibeus bats with Tacaribe virus, even in surviving bats (65). To date, no direct comparisons of infections with bat-borne viruses in reservoir host bats and pathology models have been performed; thus, there are no direct comparisons of the antibody responses to determine differences or similarities between bats and other mammals. In addition to immune response studies of apathogenic infection of bat reservoir hosts and their viruses, it will be necessary to examine the immune response in pathogenic infections of bats for essential comparison, such as the aforementioned Tacaribe virus infection of artibeus bats (65) or rabies virus infection of bats of many species (60, 66). After all, if bat IFN responses are “always on,” why does Tacaribe virus kill bats?

## Has Persistent IFN Activity in Bats Driven the Evolution of Viruses Pathogenic to Humans?

With persistent activation of the IFN response in bat cells, it is reasonable to assume that viruses hosted by bats have evolved finely tuned countermeasures to dampen the response in the reservoir bat species. For example, STAT1, an essential component of the type I, II, and III IFN receptor signaling pathways, is a target of ebolavirus VP24, Marburg virus VP40, Nipah virus V and W, and SARS-CoV ORF6 (67–71). Because the viruses have evolved in bats, these proteins are likely optimized to disrupt STAT1 activity in the reservoir host bats in a qualitative and/or quantitative manner that permits virus replication and shedding without compromising the health of the host. Hendra virus antagonizes IFN production and signaling in an immortalized cell line from the Australian black flying fox, but only disrupts IFN production in an immortalized human cell line (72, 73). The impact of these proteins on STAT1 in the reservoir bats must be enough to allow some, perhaps periodic, viral replication to sustain the virus in bat populations, but not so much that it leads to high levels of viral replication and shedding from infected cells; otherwise, pathology and/or a robust adaptive immune response would ensue. But because the IFN receptor pathway is persistently activated in bat cells, it is likely that expression of these viral proteins occurs to counteract STAT1’s cascading activation of the ISG pathways. Another important caveat of the experimental systems used to examine the effects of these viral proteins is that their genes are often cloned into high-expression plasmids or into other viruses that do not naturally infect bats. It is probable that expression levels of these genes by their viruses have been evolutionarily optimized for the reservoir bat species. But this presents logistical difficulties because most of these viruses require BSL-3 or BSL-4 containment, a significant hurdle for many investigators.

**Figure 2:**
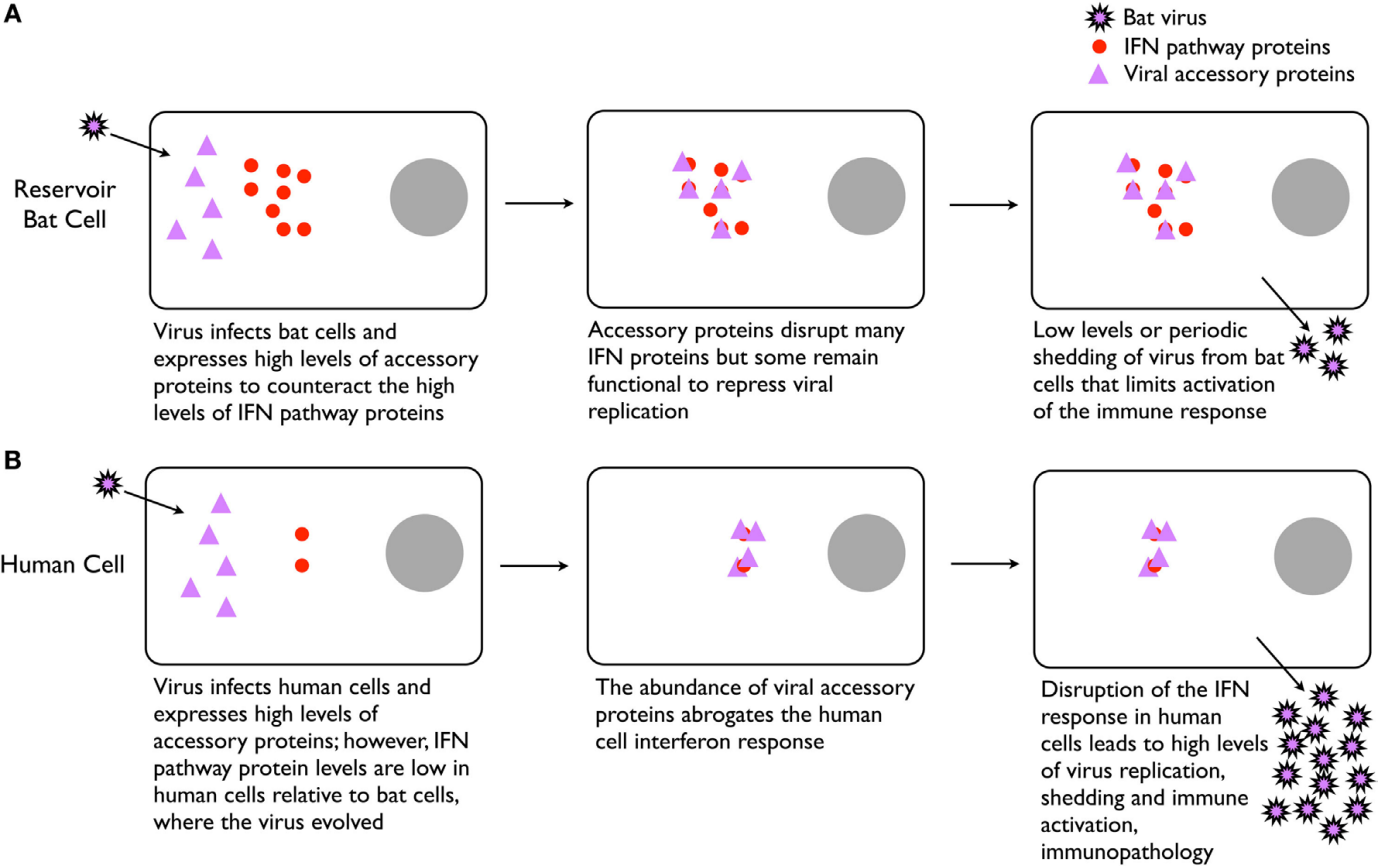
Potential explanation for high virulence of certain bat-borne viruses in humans. **(A)** Infection of bat cells leads to high expression of viral accessory proteins that repress the constitutively active type I interferon (IFN) system, leading to low levels of virus replication and shedding. Low level or intermittent replication of virus delays and reduces stimulation of the immune system, thus resulting in weak adaptive immunity and poor antibody responses. **(B)** In human cells, the high expression of viral accessory proteins significantly disrupts the cell’s ability to control the infection, leading to high levels of virus replication and immune stimulation that contributes to pathogenesis.

STAT1 is a highly conserved protein and the viral accessory proteins that target it have been shown to often interfere with human STAT1 activity (67–71). We hypothesize that because of persistent IFN activity in bat cells, these viruses may express these accessory proteins at substantially higher levels to counter the bat cell’s elevated basal levels of the IFN response genes (Figure 2). STAT1 of the Australian black flying fox (Hendra virus reservoir) and the Egyptian fruit bat (*R. aegyptiacus*, Marburg virus reservoir) are 96% and 97% identical, respectively, to human STAT1. Because the human IFN system is “off” (i.e., low basal levels) until an infection, it may be that these viral proteins are expressed in such high abundance immediately upon infection of a human cell (because they have been evolutionarily programmed in bat cells) that they abrogate the cell’s ability to mount an effective IFN response. This could lead to abundant viral replication and shedding from human cells, which would then disseminate and infect other cells, leading to direct viral pathology or immunopathology from the subsequent activation of immune cells that respond to viral infection, including macrophages, neutrophils, NK cells and cytotoxic T cells. Alternatively, or in conjunction with, the viral proteins may bind to STAT1 of humans and bats with different affinities that could contribute to the dichotomous outcomes (72).

## Caveats and Challenges of the Hypothesis

We recognize that this hypothesis on its own may be insufficient to explain the biological relationships and interactions of hundreds of viruses, and probably many more (74), and bats of the ~1,200 different species. Is the IFN system “always on” for all 1,200 species of bats? Do they all have more V(D)J germline gene segments? Do they all rely more on combinatorial diversity and less on SHM for the generation of their immunoglobulin repertoires? Just as there are significant differences between bats and other mammals, there are likely significant differences between bat species. However, considering the current evidence, we believe that several aspects of these hypotheses can be experimentally addressed with appropriate animal studies. We also have not considered other aspects of immune systems that may be important in chiropteran immunology, including activities of cellular immunity, the roles of complement and antibody-dependent cell cytotoxicity, immune effector molecules such as cytokines, antigen processing and presentation, immunological memory, and the myriad other immunological factors. Virtually nothing is known about these aspects of bat immunology, thus it is difficult to imagine how they might be different or contribute to bats of a particular species being suitable reservoir hosts for viruses of a particular species. It also is likely that many other differences between bats and other mammals exist that are not directly related to the immune response (e.g., metabolism, physiology, hormonal changes, behavior, “flight as fever”) that are contributory factors to reservoir host status of bats.

Of what is known, experimental approaches that examine the responses of infected bat cells (e.g., IFN response) and antibody responses seem to be the most tractable. A significant hurdle to accomplish these experiments is the lack of well-defined bat models for infectious disease research. Few closed colonies of bats are available for such purposes and of those, few reagents and methodologies have been developed to exploit them. These deficiencies can be rapidly overcome using the technological tools available today. For example, collection of low abundance antibodies (i.e., IgM, IgA) or immune cells from bats is challenging; most microbats are so small that collection of a few hundred microliters of blood can be lethal. But with deep sequencing, full genomes and transcriptomes can be rapidly generated and exploited to produce nearly any recombinant bat protein for use in experimental work. This approach could be deployed to generate monoclonal antibodies specific to not only IgM and IgA, but IgG subclasses, to help understand immune responses in bats. Moreover, with long-read RNA-Seq and bioinformatics, characterization of immunoglobulin and T cell receptor repertoires can identify increased frequencies of V(D)J usage during B and T cell clonal expansion and SHM during infection (75, 76). This should clarify to what degree SHM is used by bats during immune responses, which has only been examined in naive little brown bats (9).

Regardless of the virus, it is essential that experimental infection studies of bat viruses should be done in bats. The use of other species, such as rodents and non-human primates, may provide information about pathogenesis, but they cannot address the biology, evolution and ecology of bat-borne viruses, and how they may emerge as human pathogens.

## Author Contributions

All authors listed have made substantial, direct, and intellectual contribution to the work and approved it for publication.

## Conflict of Interest Statement

The authors declare that the research was conducted in the absence of any commercial or financial relationships that could be construed as a potential conflict of interest.
